# Indents

**Published:** 1904-12-15

**Authors:** 


### INDENTS.

☞ Brief Articles of Technical or Scientific Value will receive notice and comment. Contributions invited.

**Tempering Instruments.** A Frenchman recommends tempering small instruments in carbolic acid, claiming that they are more elastic and pliable than when tempered in water only.

**Tooth Powder.**

℞ Pulverized borax ℥ ij

Precipitated chalk ℥ iv

Powdered myrrh

Powdered orris root aa ℥ j

Thoroughly mix and sift through a fine sieve: color and flavoring can be added as desired.

**Bands in Regulating.** A nickel rolled out to the proper thickness serves the purpose far better than German silver. It is better in color, and remains so; is somewhat stronger, and can be soldered with the greatest ease.—*Brief.*

**Lead Washer For the Hypodermic Syringe.** Use thin lead washer in the needle joint of you hypodermic syringe—it will make a water-tight joint and does not have to be removed for a long time; besides it makes an aseptic joint.—*Dental Hints.*

**Diagnosing Devitalized Pulps.** Dr. Schamberg recommends placing the rubber dam on each of the suspected teeth, and then testing with application of concentrated heat and cold. If these are uncertain, test by trans-illumination and the radiograph. Dr. James Truman recommends the galvanic current as the most accurate method in his experience.—Discussion in the November *International.*

**Expansion of Plaster.** Dr. P. B. McCullough, in the *International*, says that if to boiled water slacked lime be added and the clear liquid decanted and is used to mix plaster of paris with, the plaster will not expand on setting. He also found that plaster mixed with gum arabic water did not expand.

**Plaster Impressions.** When a plaster impression is to be taken of a mouth, especially when some teeth remain in it, and it is free from thick saliva, it should be rinsed with a saponaceous wash to prevent parts of the impression material breaking away and sticking to the dry mucous membrane.—*Journal of Science.*

**To Renovate Hardened Mouldine.** Place in a glass and cover with water to which a half teaspoonful of glycerin has been added. Leave in a warm place until the water evaporates. The water dissolves the lumps and the glycerin thoroughly permeates the mass; a little kneading will make it as good as new.—*Dental Digest*

**Roentgen-Ray Dermatitis.** A case is reported from Langenbeck's Clinic, Berlin, in which a physician, who had used the Roentgen rays a great deal for three years, had a chronic ulcer develop at the base of one of his finger nails. The pain became so intolerable that the finger had to be amputated about two years after it developed.

**Hydrogen Peroxide Contra-indicated.** Dr. T. W. Brophy, in the November *Review*, says hydrogen peroxide is one of the most pernicious remedies used in the treatment of suppurative conditions of the jaws. He cites cases to illustrate its tendency to extend infection and produces extensive necrosis. It acts by the efferves-cence to separate the structures and to diffuse infection. It is so evanescent that it carries no antiseptic value and consequently, does not even disinfect its own destructive action.

**Argyrol.** Dr. Brophy recommends a twenty-five percent solution of this agent for the treatment of aphthous ulcers of the mucous membrane in the mouth. Carefully dry the ulcer and apply with pledget of cotton. It is disinfectant, astringent and somewhat cauterant. One application will ordinarily be sufficient. It is also a good disinfectant and antiseptic for other septic affections.—*Review.*

**Menthol Crystals.** Sometime in operating for pyorrhoea the tissues are sensitive. It may not be advisable in a given case to employ cocain on account of contra-indications. In such cases pack the pyorrhoea pockets with menthol crystals a few minutes in advance of the operation. It will be found a great relief to the patient, and is in every way a more pleasant application than cocain.—E. D. in *Review.*

**Sultan with Diamond Teeth.** The most notable figure nowadays in the West End is the Sultan of Lahore, who is stopping at the Hyde Park Hotel, London. He is constantly seen driving out in his automobile. The machine is glorified with gorgeous crests and coats of arms, but it is the owner himself who is always the center of general attraction. The reason is that the Sultan possesses a unique set of teeth, all his own, all the front ones being set in diamonds encircled in gold, and the effect at close quarters is astonishing.

**Local Anesthetics.** All operations in the mouth should be preceded by the application of a local anesthetic. Nothing acts so nicely as two percent finely-powdered cocain added to one ounce of *original package Campho-Phenique.* This produces a saturated solution, which, when applied on a large sized pledget of cotton to the entire mucous surface of the mouth, completely obtunds local sensation and prevents pain incident to removing calculus, polishing teeth, wedging, removing loose roots, and prevents gagging and nausea in taking impressions, applying the dam and ligating the same.—B. L. Thorpe, in *Review*.

**An Interesting Item.** To prevent the adhesion of soft cement to instruments while operating, keep in a convenient place a small vessel, similar to the old-style salt-cellar, filled with violet talcum powder. Occasionally insert the instrument in use into the powder and it will effectually prevent the adhesion of the cement to the instrument. Any talcum powder will be found useful, but "violet" is preferred on account of the pleasant odor. The same is applicable in working gutta-percha.—Grafton Munroe in *Review*.

**A Veteran.** Dr. John B. Rich, whose handsome portrait is printed in Sunday's New York *Sun*, was from 1836 to 1898 the fashionable dentist of New York. Now he is the president of the Microscopical Society and of the Hundred Year Club, the author of important scientific works, and, at ninety-four years of age, a marvel of manhood, his mental faculties unimpaired, his body erect and his eyesight so good that he never uses glasses except for reading. His recipe for longevity is cheerfulness, moderation in all things and a little exercise every day.

**Adaptation of Partial Denture To Remaining Teeth.** When only one or two teeth remain, as the two upper canines for instance, a closer adaptation to the teeth may be secured by slightly trimming the plaster teeth and completely encircling them with soft or velum rubber. Pack the ordinary rubber around this and vulcanize as usual. In finishing use a sharp knife with both the knife and velum rubber wet. A snug adaptation will be attained, with a support superior to that given by clasps, and less harmful to tooth-structure.—P. B. McC., in *Inter. Dental Journal*.

**A Short Rule for Determining Percents in Mixtures.** Multiply 480 by the percent desired and point off two right-hand figures. The figures at the left of separatrix will give the number of grains or drops; 480 is number of grains to the ounce. Examples: 480 × 4 = 1920; 19.20 = 19 1-5; 19 1-5 grains to an ounce of liquid, or 4 percent solution. 480 × 10 = 48.00, or 48 grains to one ounce—10 percent. The above may have been published previously, but I have never seen it. I have been asked so often how to find the percent that I think this may be of interest.—A. C. Hewett, in *Review.*

**Tooth Powder.**

A good formula for a tooth powder is the following:

| | | |
|---|---|---|
| Precipitated chalk | grammes | 500.0 |
| Menthol | gramme | 0.5 |
| Oil of eucalyptus | Cc. | 0.5 |
| Oil of wintergreen or cassia | Cc. | 4.0 |
| Saccharin | gramme | 1.0 |
| Powdered Castile soap | grammes | 4.0 |

Mix the menthol and the oils before adding the other ingredients. Color with carmine, two grammes to 500 grammes of chalk, if a pink color is desired. Put up in regular tooth powder style. Costs from five to ten cents, according to the size of bottle.—*Bulletin of Pharmacy.*

**Method of Making a Gold Inlay.** Burnish a platinum matrix as for a porcelain inlay, paying no heed to small openings in the platinum. Paint the surface of the matrix that comes in contact with the tooth to be restored, and all parts not intended to be covered with gold—where the matrix extends beyond the margins—with a thin mixture of prepared chalk and water. The matrix is now filled with the required carat of gold and the gold fused, using no flux. The gold will now flow beyond the margins. Set small inlays before removing the surplus matrix and allow the cement to set before finishing.—F. W. Stephan, in *Review.*

**Tooth Resorption.** Dr. Schamberg reports in the November *International* a case of resorption of the roots of live permanent teeth. A peculiar swelling appeared over a live central incisor tooth. There was no abscess and a radiograph showed considerable absorption of the root. After several weeks other teeth became involved, and there was so much absorption of the first tooth that its crown came off. The patient was about thirty-five years old and there was no indication of pyorrhoea. There was some absorption of the process, probably due to the ingress of infection material from the mouth.

**Odontalgia.** Toothache, which may not be controlled by local applications, can be many times very satisfactorily treated with systemic anodyne remedies. The combination of antikamnia and codein, in tablet form, supplies a convenient remedy. The five-grain tablets contain four and three-fourths of a grain of antikamnia and one-fourth grain of codein. A tablet given shortly previous to a painful operation, or to control the after pain from extraction, is very useful. Like all good and powerful remedies, this one should be administered intelligently and always under the direction of the dentist.

**A Simple Retaining Appliance.** To retain an incisor tooth corrected from a torsal relation or mal-alignment, take a piece of 34-gauge pure gold plate and adapt it to the entire lingual surface and extend over incisal edge for a sixteenth of an inch. Cut away the central portion of the laps down to the incisal edge, leaving two straps near the mesio and disto-incisal angles to extend over slightly on to the labial surface. Then burnish the backing to a close fit on the labial surface of the tooth, and solder to its lingual surface a spur which will serve to attach it to a vulcanite plate, using enough solder to stiffen the backing and prevent distortion.—Dr. Windell, in *Office and Laboratory*.

**The Protective Role of Human Saliva.** Dr. Miller, of Berlin, finds that the saliva of man, *per se*, possesses absolutely no protective or antiseptic properties. The sulfocyanid of potassium, likewise, has no bactericidal action, nor has the buccal mucosa. The saliva contains no cytoses and is not hemolytic. The secret of the antisepsis of the buccal cavity lies in the buccal flora. By simple overgrowth they crowd out and eliminate the majority of saprophytes and pathogenic bacteria. In the diseases of the soft parts, phagocytosis plays an important role. Caries is very largely predetermined by the character of the teeth.—*Med. News.*

**Using Tooth Powder.** I ask my patients how many times they use a powder when they are brushing their teeth—how many times they put powder on the brush while cleaning the teeth; and nine out of ten will tell you that they simply put the powder on the brush once and brush their teeth. I tell them to use it several times. They certainly cannot get around all the teeth with one brushful of powder. Now, the directions which I give to patients are, that they will first put powder on the brush for ne side, and put on more powder to brush the other side, and then more for the masticating surfaces, until they have reached every portion of the mouth with powder and brush.—A. C. Rich, in *Cosmos.*

**Preparing an Impression Tray for Plaster.** To prevent the plaster from flowing down the patient's throat when taking an impression, prepare the tray as follows: Puncture the impression tray near the heel, making several holes about a quarter of an inch in diameter. Build up the heel with wax to reach the palate, and from this carry a piece of sheet wax forward under the tray. When taking the impression the surplus plaster will be forced through the holes and carried forward by the sheet of wax under the tray, thus effectually protecting the throat. The same result may be attained by cutting a V-shaped piece from the rear of the tray and building with wax, as described above.—F. W. Stephan in *Review*.

**Rubber Cement.**

I.

| | | |
|---|---|---|
| Gutta-percha, in pieces | ounces | 2 |
| Carbon bisulphide | ounces | 4 |
| Oil of turpentine | ounces | 1 |
| Asphalt, in powder | ounces | 2 |

II.

| | | |
|---|---|---|
| Gutta-percha | parts | 2 |
| Caoutchouc | parts | 4 |
| Fish glue | parts | 1 |
| Carbon bisulphide | parts | 26 |

III.

| | | |
|---|---|---|
| India rubber | grains | 15 |
| Mastic | grains | 240 |
| Chloroform | ounces | 2 |

Dissolve the rubber in the chloroform and add the mastic. Let stand for a week or two before using.—*Druggists' Circular*.

**Value of Exercising The Teeth.** "Tom Bowling" writes to the Glasgow *Record*: "Some forty-five years ago I joined the Royal Navy as a second class boy, fourteen years of age, and lived principally on salted provisions and hard ship's bread for seven years, when I was compelled to leave through an accident. I am now in my sixtieth year and have thirty sound teeth, white and hard in my gums, and I attribute this to being compelled to eat hard food, which required a great deal of mastication, in my youth. My opinion is that children are fed too much on slops, such as soups and stews, and that the teeth do not get enough to do and, as is well known, if any part of our bodies does not get sufficient work nature causes that part to atrophy. I've put away a great deal of salt in my time and believe it to be beneficial. My idea of a remedy is to give youngsters plenty of hard food to chew and nature will do the rest."—*Record*.

